# Awareness, Utilization and Perception of Sexually Transmitted Infections Services Provided to Out-of-School-Youth in Primary Health Facilities in Tshwane, South Africa

**DOI:** 10.3390/ijerph20031738

**Published:** 2023-01-18

**Authors:** Boitumelo Ditshwane, Matilda M. Mokgatle, Oluwafemi O. Oguntibeju

**Affiliations:** 1 School of Public Health, Sefako Makgatho Health Sciences University, Pretoria 0208, South Africa; 2 Department of Biomedical Sciences, Faculty of Health and Wellness Sciences, Cape Peninsula University of Technology, Bellville 3575, South Africa

**Keywords:** sexually transmitted infections, perceptions, attitudes, facilities, youth, behavior

## Abstract

Background: Despite the availability of different health care initiatives and interventions, young people are still faced with barriers in accessing reproductive health care services; thus, they are exposed to health-related issues such as sexually transmitted infections. Aim: To determine the awareness, utilization and perceptions about sexually transmitted infections services provided to out-of-school-youth in primary health facilities in the Tshwane district, Gauteng Province, South Africa. Methods: The study employed a quantitative, cross-sectional descriptive survey with a sample size of 219 to determine the level of awareness, utilization and perceptions about sexually transmitted infections services provided to out-of-school-youth in Tshwane district. Results: Out-of-school-youth between the ages of 18–24 years participated in the study. Most of the participants (90.8%, n = 199) were female. Service utilization was high in females compared to their male counterparts. There is availability of youth-friendly services in primary health care facilities, however, the level of service utilization among young people is still a challenge evidenced by 12.1% (n = 74) of participants who never sought treatment for STIs, although they had STI symptoms. Furthermore, 52.0% reported that they were not happy with the health services they received when they had STIs. These findings clearly indicate a gap in service delivery for young people regarding reproductive health issues; thus, the low health care seeking behavior among the youth. Condom use was 69.1% and/or inconsistently used among the youth; about 80% of the participants had low perceptions of the risk of contracting STIs. The self-reported risks of HIV and AIDS was 46.8%. Approximately 20% reported that they would not refuse to have sex if their partner did not want to use condoms. These findings showed risky behavior among the participants, and shows that the level of awareness about the risk of contracting STIs is still poor. Conclusions: Irrespective of facilities with youth-friendly services, out-of-school-youth still display poor perceptions about sexually transmitted infections services due to health care providers’ attitudes, limited resources, and working hours. Furthermore, the level of awareness regarding sexually transmitted infections is poor, hence the display of risky sexual behaviors.

## 1. Introduction

The risk of contracting sexually transmitted infections (STIs) among adolescents and youth remains a problem, due to risky sexual behaviors [1]. Globally, STIs are regarded as a public health concern [2]. Young people under the age of twenty-five years account for 41% of new HIV infections globally [3,4]. According to Godia et al. [5], young people in sub-Saharan Africa experience an increased risk of sexual and reproductive health problems. Studies conducted in South Africa have also shown that the country has the highest prevalence of STIs and HIV among young people, with Kwa-Zulu Natal having the highest among the nine provinces [6,7]. In Tshwane district of the Gauteng province, South Africa, the rate of HIV/AIDS among youth between the ages of 15–24 is 19.3% in females and 5.7% in males [8].

Globally, health care services, particularly sexual and reproductive health (SRH) services, have been designed to be youth-friendly to ensure that the youth find the services easily and they are acceptable to access [1]. Although countries have taken initiatives to ensure that the health care needs of youth are taken into consideration by making services user-friendly, there are still places that are not youth-friendly [9]. 

The South African Department of Health [8] adopted the Love Life’s youth-friendly services as a national policy in 2006; however, studies conducted in Gauteng and KwaZulu-Natal have shown that youth are not utilizing sexual and reproductive health services as expected. Studies have shown that despite the youth being aware of the STI services rendered at facilities, there is still A low use of these services due to barriers, such as a fear of being judged or scolded, or poor quality of care and chastisement by health workers for being sexually active [1,10]. Moreover, the South African Department of Health has put in place many initiatives and developed policies with the aim of promoting the wellbeing of youth, addressing issues that affects the health of young people between the ages of 10–24 years, as well as ensuring that resources such as sexual health care services are available, user-friendly and accessible to them [8].

A literature report has shown that out-of-school-youth are at the higher risk of acquiring STIs compared to those who are still in school, this is due to limited access to health education and information [11,12]. Studies conducted in South Africa have shown that the uptake of STI services by youth remains relatively low [1,10]. Few studies have been conducted among youth in South Africa in general, and there is a paucity of research about out-of-school-youth, who are at high risk of contracting HIV and other STIs. Furthermore, research has shown that there is a low utilization and awareness of STI services in South Africa [8]. A gap in the literature also shows there is limited research on the assessment of acceptability and the appropriateness of reproductive health services among out-of-school-youth, hence the conceptualization of this study and the motivation for the study. It is our opinion that study would help to bridge the gap in the level of awareness and provide more information for public health interventions aimed at improving STI service uptake.

## 2. Materials and Methods

### 2.1. Study Design

The study employed a quantitative, cross-sectional descriptive survey among out-of-school-youth in Tshwane district clinics. The quantitative research method was used because the participants and the variables that were studied were quantifiable [12,13,14]. A cross-sectional design was used because the authors wanted to gather information about the relationship between variables at one point in time [15]. The descriptive quantitative survey was employed to determine the awareness, utilization, and perception about sexually transmitted infection services provided to out-of-school-youth.

### 2.2. Study Setting 

The study setting refers to the place where the data were collected [15]. Tshwane is a district in the Gauteng province with seven sub districts namely sub district 1 to 7. The total estimated population of Tshwane is 3 275 152 and it is the administrative capital city of South Africa. Sub district 1 is an urban area located in the north side of Pretoria. The most common spoken languages are Setswana, Sesotho, Sepedi, Xitsonga, Tshivenda, English and Afrikaans. There are 25 health care facilities and 35 ward-based outreach teams in sub district 1, three community health centers, one district hospital, one central hospital, and 18 clinics [8]. Some of the primary health care clinics in Tshwane fall under a local municipality and the rest fall under the Gauteng provincial government. Clinics operate from 07h30–6h00 whereas others are operating 24 h as community health centers. These clinics are within a five-kilometer radius and the most common modes of transport used in the community are taxis, buses and trains. The choice for the research setting was informed by health facilities that have youth-friendly services where out-of-school-youth can be easily accessed by the research team.

### 2.3. Study Population

For this study, the population was out-of-school-youth aged between 18–24, males and females, utilizing the youth-friendly services in Tshwane district 1 health care facilities. The out-of-school-youth in this study are defined as those who dropped out-of-school and those who could not pursue their studies further.

### 2.4. Sample Technique and Sample Size

A convenient sampling technique was used to select the study participants. This technique was used for sampling because it enabled everyone meeting the criteria to be part of the study. For this study, all out-of-school-youth aged 18–24, males and females, who visited the facilities were recruited into the study. On average, the number of clients who attend youth-friendly services in Tshwane sub district 1 is 300 per month, with an annual total estimate of 3600 patients. For this study, the sample size was calculated using the Raosoft (Raosoft Inc., Seattle, WA, USA) sample size calculator and calculated to be 348. The sample size was determined based on the estimated total population of 3600 youth attending the youth-friendly services centre in a year at a 95% confidence interval with a 5% margin. The proposed sample size was 348, however, there was a challenge recruiting “out-of-school-youth” aged 18–24 at the facilities, which led to the decreased sample size.

### 2.5. Inclusion and Exclusion Criteria

Out-of-school males and females, aged 18 to 24 years, visiting the youth-friendly facilities and who volunteered to participate in the study and gave consent, were included in the study. School goers and the out-of-school-youth that met the criteria, but who did not consent to take part in the study, were excluded.

### 2.6. Recruitment

Recruitment started after ethical clearance was obtained from Sefako Makgatho Health Sciences University Research Ethics Committee (SMUREC: /H/41/2018: PG) and permission was granted at the Tshwane Health District (GP_201803_001). The research team scheduled appointments with the nursing sisters in charge of the youth-friendly facilities to seek permission for data collection and to explain the purpose of the study. The recruitment process started from August 2018 and continued until January 2019. The participants were recruited, conveniently, in the waiting area of the youth-friendly facilities where the purpose of the study was explained to them. Participants who agreed to take part in the study were taken to a private consulting room, where they signed the consent form. 

### 2.7. Data Collection and Collection Tool

Data were collected using a structured questionnaire. Data collection took place at three youth-friendly facilities in Tshwane Health District during clinical operating hours on weekdays, until the required sample size was achieved. The questionnaires were designed in Setswana, Sepedi, and English. The questionnaires were guided by research objectives and adapted from a standardized questionnaire [16]. The questionnaire had five sections: the first section required information about the self and the family; the second section was about condom use; the third section was about HIV risk perceptions; the fourth section was about knowledge of STI services; and the fifth section was about the utilization of health services. It was proposed that data should be collected by using self-administered questionnaires, however, the participants were not comfortable to administer the questionnaires themselves. Therefore, the research team administered the questionnaires for the participants after they had signed the consent form and only English questionnaires were used. It took 15–20 min to complete each questionnaire. 

### 2.8. Data Analysis

Data analysis was performed to turn the raw data collected into meaningful data in order to ensure that the research questions and objectives were answered [14]. Quantitative data analysis was appropriate because the research findings could be generalized to the larger study population [14]. Raw data was captured using a Microsoft Excel spreadsheet, cleaned, coded, and checked for quality and then imported into STATA software version 13 (StataCorp, College Station, TX, USA) for analysis. Descriptive statistics, using frequency distribution, were used to analyze the categorical variables. The confidence interval (CI) was set at 95% and the *p*-value set at 0.05%. The data were presented using tables and bar charts. Tables are suitable because they summarize the findings in a meaningful way and enable easy understanding of the results.

### 2.9. Validity and Reliability 

A pilot study of 20 participants was conducted at the youth-friendly facilities to pre-test the tool for validity and reliability. To ensure validity and reliability, a standardized tool was used throughout the data collection and the authors received the same responses from participants each time the data were collected.

### 2.10. Ethical Consideration 

Ethical clearance was obtained from the School of Health Care Sciences Research Committee (SHCSRC) and Sefako Makgatho Health Sciences University Research Ethics Committee (SMUREC). Permission to conduct the study was also obtained from Tshwane District Health Research Committee and the clinic managers of the youth facilities. Voluntary participation was maintained throughout the data collection and informed consent was obtained from the participants prior to data collection. Anonymity was ensured by not gathering any personal information from the participants, such as their names and addresses. Participants’ confidentiality was always maintained and no names or any personal information was written on the questionnaire. The information collected was kept in a locked cupboard that was only accessed by the research team.

## 3. Results

The analysis was conducted at a 95% confidence interval as in any social science analysis. It provided descriptive statistics for sexual behavior, perceptions of HIV, condom use, STIs and health seeking behaviors. It proceeded with testing the associations between STIs and sexual and reproductive health factors. A regression analysis was applied to check if any of the factors had a relationship with STI occurrence. 

### 3.1. Socio-Demographic Characteristics of Participants

**Figure 1:**
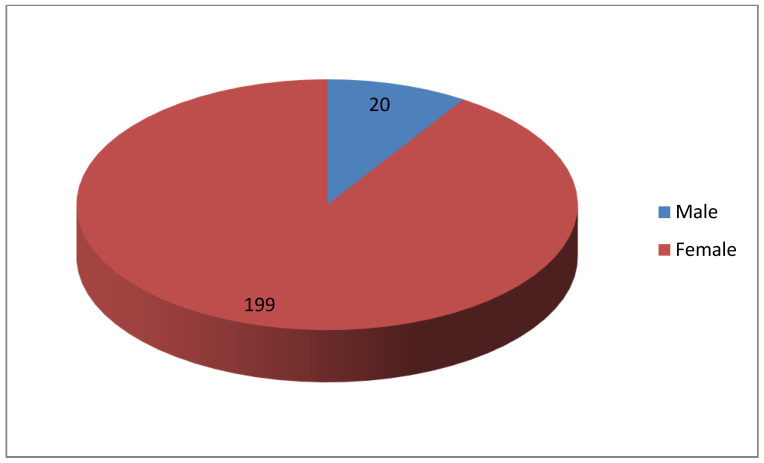
Demographics (gender) (n = 219) of participants.

Figure 1 shows that most of the participants were female.

**Figure 2:**
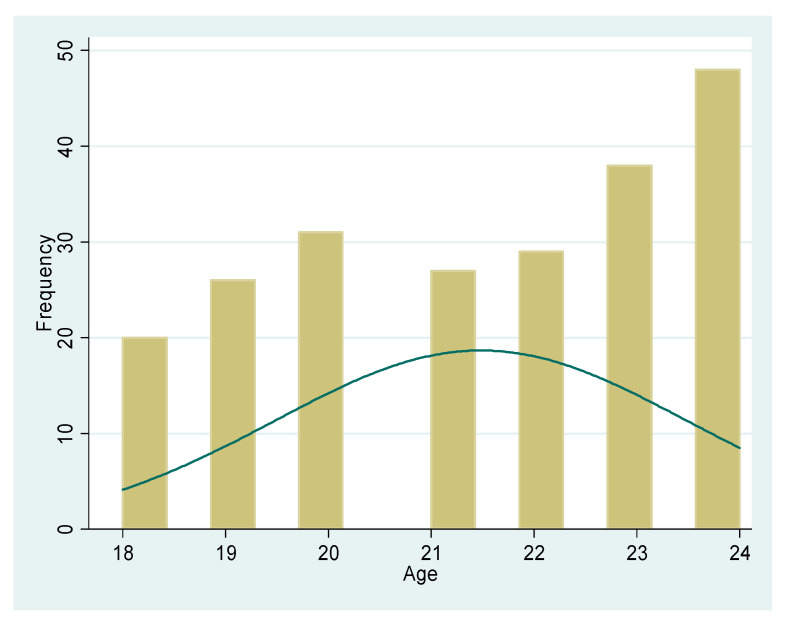
Age distribution of participants.

Figure 2 indicates the participants’ age, ranging from 18–24 years, with a mean age of 21.4 years.

Table 1 indicates that three-quarters of the participants (74.8%) travelled less than 5 km to reach the clinic or facility (the clinic or facility was within a radius of 5 km). A fifth (21.9%) of the participants travelled 5–10 km, while very few lived more than 10 km away from the clinic or facility (3.2%). The time to it took to reach the clinic was less than 30 min for 88.1% of the participants. A tenth (11.8%) of the participants travelled for 30 min to an hour. At least seven out of every ten (73.5%) walked to the clinic, and a fifth (21.4%) used a taxi, while very few (4.57%) used own transport.

**Figure 3:**
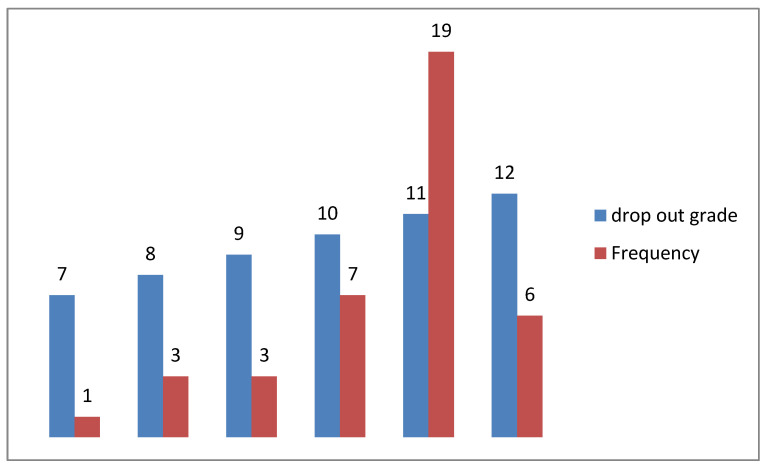
Drop out grade (n = 39) of participants.

Figure 3 indicates that at least 19 ouf of 39 participants dropped out in grade 11 and 6 dropped out in grade 12. A quarter of the participants dropped out of school before grade 11. 

Table 2 indicates that the majority of the participants 91.7% (n = 201) had had sex while a few had never had sex. The median age of a first time sexual experience was 17 years, with at least half who had experienced their sexual debut when they were between 13 and 17 years (adolescents); and 47.9% experienced their sexual debut when they were 18–24 years (post adolescent stage). Three-quarters (76.0%) of participants indicated they had planned their first sexual experience, while up to a fifth (23.5%) had an unplanned first sexual experience. Approximately six out of every ten (57.1%) participants had sex with partners who were older than them, while at most, three out of every ten (28.3%) had sex with partners of their own age. Very few had sex with partners younger than them. Six out of every ten (60.3%) participants indicated that when they first had sex, they were both in school with their partner, indicating that there was sexual activity happening in schools from a very young age. Almost a quarter of them (23.3%) indicated that they experienced first time sex with a partner who was working, while very few experienced first time sex with a partner who was not attending school.

Table 3 indicates that seven out of ten (72.6%) were in a current relationship, while up to a quarter were not in any relationship. Almost nine out of ten (89.9%) participants had a steady partner, while a tenth (10.6%) had a casual partner. Further results indicate that eight out of ten (82.3%) participants had current partners who were older than them, while very few had partners who were their age (8.81%) or younger than them (8.81%). Two-thirds (66.2%) of the participants indicated that their current partners were employed, while a few were unemployed (15.9%) and 17.8% were attending school. Seven out of ten (72.5%) participants had been in a relationship for more than 2 years, while a quarter (25.1%) had been in a relationship for 1–2 years; only 2% were in a relationship for less than a year.

Eight to nine out of ten participants (Table 4) had had one partner in the 12 months preceding the survey (88.5%). The participants were more likely not to have had one partner at a time (81.7%) in their lifetime, and were more likely not to have had more than one partner in the 12 months preceding the survey. Eight out of ten (81.7%) participants reported not having more than one partner at the time of the study. Most participants had never had sex in exchange for money (98.5%) and had not done so in the six months preceding the survey (99.5%). Many participants had never had casual sex with someone who was not their regular partner (82.4%), and the vast majority had never done so in the 6 months preceding the survey (95.2%). These results show that 16.5% of participants had had sex with someone who was not their regular partner, and very few (4.74%) had done so in the past 6 months.

Table 5 indicates that a tenth (12.6%) of participants had drunk alcohol the last time they had sex, and over three-quarters had planned for it (79%). This leaves eight to nine (86.8%) out of ten who had never drunk alcohol, and a fifth (20.9%) who had unplanned sex. Two-thirds (67.8%) of participants had been pregnant or impregnated a girl (42.3%); girls were more likely to state they had been pregnant than boys advise they had made someone pregnant. Most participants had discussed contraception (90.3%), and 77.5% had used contraception of some sort in the past 6 months. This translates to a fifth of participants who had never used contraception, amid two-thirds of girls who were likely to be pregnant.

**Figure 4:**
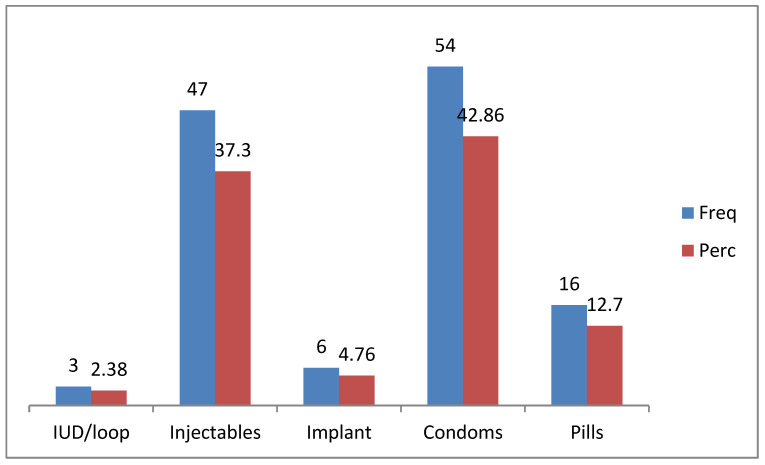
Contraceptive methods (n = 126) used by the participants.

Figure 4 illustrates the types of contraceptives used, which include condoms (42%), and injectables (37%). This suggests that three to four out of every ten participants were using condoms and any type of injectables. Very few were using implants (5%), or loop (2%), while a tenth (12%) were using pills. Three-quarters (74.5%) of participants obtained their contraceptives from clinics or hospitals, while a quarter (25.4%) bought them from shops/pharmacies.

Four out of every ten (83.5%) participants perceived low chances of falling pregnant, while a few perceived high chances (16.4%). For boys, five to six (57.8%) of participants indicated that the chances of making a girl pregnant were unlikely, while a third perceived they would likely impregnate a girl (Table 6).

The majority (99%) of the participants indicated that male condoms were easily available for the youth in their community. Eight out of ten (81.2%) females indicated that female condoms were easily available for them in the community. Eight out of ten (81.2%) felt they could purchase condoms without feeling embarrassed and could go to obtain condoms from a public place without feeling embarrassed. Six to seven (68.9%) participants indicated that they always carried condoms with them should they need to use one, while three out of ten (31%) did not always carry condoms. Nine out of ten (90.4%) participants felt confident suggesting the use of condoms with a new partner, while eight out of ten (82.6%) indicated they could refuse sex if their partner did not want to use condoms (Table 7).

Almost all of the participants (97.2%), except for six, self-reported that they had tested for HIV. The majority (91.6%) had tested in the past 12 months. Eight out of ten (82.1%) indicated they knew the current status of their partners (Table 8). The majority (90.6%) indicated that they did discuss HIV testing with their current partner, while (85%) indicated that they were likely to ask their current partner to go for an HIV test.

Table 9 indicates that six out of ten (59.2%) participants felt that a person can have an STI without symptoms in the early stage of the STI, while a third were uncertain whether this was true or not. Eight out of ten (79.7%) participants perceived a low chance of getting an STI, while a fifth (19.1%) perceived a high chance. Four out of every ten (46.8%) were not worried about contracting HIV/AIDS, 30.9% expressed they were very worried, while a fifth (22%) were worried. This suggests that over half of the participants were worried about contracting HIV/AIDS. This should be worrisome considering the high HIV levels among adolescent girls and young women in SA.

As shown in Table 9, at least half (51.4%) of the participants reported the use of a condom the last time they had sex, while the other half (48.5%) had not used a condom. Reasons cited for not using a condom were: a condom was not available; did not plan to have sex; I do not like condoms; my partner does not like condoms. At least four out of every ten (46.4%) participants reported that they sometimes used condoms in the last 6 months, 30.8% always used condoms, and a fifth (22.7%) had never used condoms in the past 6 months. Many females were unlikely to have used female condoms in the last six months (95.1%). The reasons cited for non-use were that these were not easily available, not easy to use, or that they had never thought about it. 

In the past twelve months, 33.8% had experienced burning (Table 10) while urinating, and/or discharge and itching in the genitals, while 64.3% did not. Of those who experienced these symptoms, 87.8% received treatment while 12.1% did not seek treatment. In total, 87.8% informed their partners that they had an STI, while 12.1% did not. When they received treatment for (burning urine, discharge and itching), the majority (84.7%) completed the treatment that they received, while a few did not (15.2%). Those who reported not completing their treatment reported that they were cured before the treatments’ completion. Eight out of ten (79.1%) participants reported that at the time that they were receiving treatment (for burning urine, discharge, itching) they abstained from sexual activities; this is in contrast with a tenth (10.4%) who indicated that they continued with sexual activity.

Reasons for going to the clinic (Table 11) were reproductive health related: pregnancy, pap smear and contraceptives (38.9%), pregnancy check-up (27%), and for consultations (10%), as well as accompanying friends or relatives (8.72%). A few came to collect ARVs (4.13%), HIV tests (9.24%), and for dental reasons (1.38%).

### 3.2. Service Satisfaction

Reasons for service dissatisfaction included: the clinic was too far, long queues and slow service, inflexible operating hours, and nurses who were rude. Furthermore, reasons why the participants felt their needs were not attended to included: doctor was not available, there was no treatment the last time they visited, they never received treatment, there was a shortage of contraceptives, shortage of medication, at times there was no medication, the staff were very slow, and that there were no pregnancy tests and injections. 

### 3.3. Availability of Services

Seven out of ten (70.1%) participants indicated that the services were available every time they visited the clinic. However, others cited their reasons for dissatisfaction, including injections for prevention/family planning being unavailable, there was a lack of staff, and a shortage of contraceptives and medication. Furthermore, there were no pregnancy tests and injections and, at times, they were told their cases were not an emergency and there was a long queue.

### 3.4. Recommendation of Services

Seven out of every ten (73.9%) indicated they would recommend the clinic to others, while a few indicated they would not. The reasons for not recommending included the attitudes of staff towards patients, bad service, inadequate services, a lack of service, long queues, and poor service delivery. In addition, the clinic was always full, the staff were very slow, not flexible, the nurses were not kind, took too much time for lunch, and they did not take things seriously. Lastly, they did not listen to people, nor did they pay any attention to clients, the service was not good, and they did not perform their duties well.

### 3.5. Service Improvement 

Seven out of every ten (73.9%) indicated that there was a need to improve the services they received, while a tenth (14.1%) felt there was no need for improvement.

## 4. Discussion

### 4.1. Demographics Findings; School Drop-Out and Age of Sexual Debut

The study sample consisted of out-of-school-youths utilizing youth-friendly facilities in three clinics at Mabopane, Soshanguve and Winterveldt in Tshwane district 1. The findings showed that at the time of the data collection, the majority of participants were female 91% (n = 219). These findings are consistent with reports in other African countries stating that females utilize health care services more frequently than males [9]. Three-quarters of participants were walking to access the clinics, with a travelling distance of less than 5 km. This walking distance of less than 5 km to access primary health care (PHC) facilities is considered fair by the South African Department of Health [8] for public health care users. The Education Department of South Africa is faced with school drop-outs, with an estimated dropout rate of 60% [17]. As observed in this study, 17.8% (n = 39) of participants dropped out of school before matriculation (before completing high school). 

Our findings showed that the median age of first-time sex was 17 years, while a few of the participants started between 13 and 17 years. The findings correspond with other studies performed in sub-Saharan countries, where both male and female youth start having sex as early as 15 years [6,9,17]. With reference to South Africa, findings have revealed that they start having sex as early as 13 years [18,19], while in Tanzania, the literature has shown that young females start as early as 9 years [20]. Considering that the target group was an older age group, from 18–24 years and out of school, most of them had already started engaging in sexual activity much earlier. These findings are consistent with a study conducted in eight African countries concerning the early sexual debut among school-attending adolescents [16,21].

### 4.2. Sexual Behaviours

Multiple sexual partners continues to be a challenge among young people in sub-Saharan countries. Findings from this study showed that 18.2% participants had more than one sexual partner at a time and these findings are alarming considering the high prevalence of STIs among young people as a result of multiple sexual partners. Similar findings of multiple partners have been reported from studies conducted in South Africa and Ethiopia [22,23]. The majority (82%) reported that they were dating older partners; similar findings from another study revealed a link between multiple partners, older partners and risky sexual behavior among school dropouts [12,24,25]. 

### 4.3. Alcohol Intake

Alcohol abuse has been associated with risky sexual behavior in sub-Saharan Africa [26]. In this study, we reported that 12% of participants never drank alcohol the last time they had sex, while 21% reported that the last encounter was unplanned, and alcohol related. Although the study did not investigate the association between alcohol intake and planned last sex, the findings highlighted the necessity to address concerns relating to substance abuse among youth. 

### 4.4. Contraceptive Methods and Use

The study reports that the most preferred method of contraceptive used was male condoms and injectables, while a few preferred pills and other methods. Furthermore, only a few used female condoms. These findings are consistent with a study that was conducted in Limpopo, and another population-based survey that was performed in South Africa, with most participants preferring male condoms compared to other methods and only a few using female condoms [27,28]. Although the majority (81.2%) of participants reported easy access to female condoms, only a few were using them, and they cited the reasons for not using female condoms as being not user friendly, not easily accessible, and some never thought about it. Similar to these findings, a study conducted in Nigeria showed that only a few participants ever used female condoms despite the majority of them knowing about them; here, the cited barriers were that they were not easy to insert and that they were expensive and unavailable [29]. Although the National Department of Health took the initiative of making condoms appealing by making them in different flavors, and freely accessible in many public places, the findings from this study showed that a quarter of participants still buy them from shops and pharmacies. Maybe this could be related to the embarrassment of obtaining condoms in public places or the avoidance of going to heath care facilities. Perhaps these findings may inform future studies that may explore the reasons for buying condoms instead of accessing them for free. 

### 4.5. Condom use and Accessibility 

The majority of participants reported easy access to both male and female condoms, as well as expressing that they could refuse sex if their partner did not want to use condoms; however, usage is still not consistent. It was observed that only 51% of participants used condoms during the last sexual encounter while 46.4% reported inconsistent use and 23% did not use condoms in the last six months. These numbers are worrying considering the high prevalence of STIs among youth. Our result is consistent with other studies that reported ineffective condom use among youth [30,31]. The reasons stated for not using condoms included that the sex was not planned, condoms were not available, and their partner did not like using condoms. A study conducted in Botswana showed inconsistent condom use and the reasons for not using them includes pleasing their sexual partners, issues of trust, and faith in their partners [31].

### 4.6. HIV Testing Practices 

We report that most of the participants (92%) had tested for HIV in the past 12 months. Similarly, findings from a study conducted in Uganda also showed a slight increase in HIV testing among youth aged 15–24 years [32]. A study performed in South Africa showed that 52.2% of youth aged 18–24 years reported testing for HIV [33], while another study conducted in Soweto in Gauteng Province showed that 47% of the participants had never tested for HIV, in a group aged 15–24 [34]. On the other hand, a study conducted in Cape Town, South Africa, by Mendelsohn et al. [35], revealed that HIV testing among youth is still low. This calls for more awareness among people about the importance of HIV testing and its benefits.

### 4.7. STI Awareness and Perceptions 

The study revealed that 53% of participants perceived a risk of contracting HIV and that they were aware of STI symptoms. These findings are similar to those of a study that was conducted among out-of-school-youth in Western Ethiopia, with 54.3% expressing a perceived risk of contracting HIV (Negeri, 2014). Almost half of the participants reported not being worried about contracting HIV/AIDS, while about 70% of them reported using condoms inconsistently. These findings show that there is a poor level of knowledge concerning STIs among young people, or that this knowledge does not necessarily translate into practice. Other studies have also reported poor knowledge of HIV among youth [32,34]. Regardless of STI knowledge and perceptions, health-seeking behavior among youth continues to be an issue [1]. Our study shows that not all youth who had STI symptoms sought health care services, and for those who sought health care services, a proportion (15.2%) did not complete their prescribed treatment, while 12% did not notify their sexual partners. Untreated STIs are a worrying matter because of the risks of infection spread and disease complications.

### 4.8. Utilization of Services

It is evident from the results of our study that young people still experience challenges with the availability of services they need, including a shortage of medication, such as contraceptives among others. Almost a quarter of the participants noted that they will not recommend the clinics to anyone because of staff attitudes, bad services, inadequate services, and long queues, among other things. Similar reasons were cited as barriers by Schriver et al. [10]. It was further pointed out by the majority (70.1%) of the participants that services can be improved by recruiting additional staff, reducing long queues, providing good service delivery, and improving staff attitudes. These findings are consistent with other studies [36].

## 5. Conclusions

The level of awareness and perceptions of STIs is still poor among youth as evidenced by their practices, which promote risky behavior and the spread of STIs. This shows that health education needs to be strengthened in order to equip youth with appropriate knowledge and behavior modification regarding their sexual health. Although young people are aware of the youth-friendly system, their service utilization level remains a challenge due to some barriers. These barriers to accessing health care services, as faced by out-of-school-youth, may also pose a limitation on their existing sexual and reproductive health knowledge and awareness. However, HIV testing is gradually improving among youth, as evidenced by the majority of participants having reported undergoing HIV testing in the past 12 months. 

## 6. Limitations

The study was conducted in one district and the sample focused only on the out-of-school-youth who had access to youth-friendly health facilities; therefore, the findings cannot be generalized to the general population of South African youth. A non-probability, convenient sampling method was used to select participants and only out-of-school-youth accessing the youth-friendly services were recruited; therefore, the possibility of response bias could have been present. The recruitment and accessing of out-of-school-youth from PHC facilities limited the reasonable distribution of genders in the sample, since it is mostly young women that access health care services compared to their male counterparts.

## 7. Recommendations

The utilization of health facilities requires sensitization and buy-ins from the health care workforce as well as adequate human resources to assist in capacity building and improved care. There is also a need to reinforce health promotion approaches to empower out-of-school-youth with appropriate knowledge that will enhance their decisions and health seeking behaviors regarding their sexual health. There is a need to ensure that young people are aware of the youth-friendly services within their health care facilities. The service should always be made available and accessible. The staff offering these services should be fully equipped with knowledge and have non-judgmental attitudes towards dealing with the needs of young people.

## Figures and Tables

**Table 1:** Travelling distance, transport and education (n = 219).

Variable	Response	Frequency (N)	Percentage (%)
**Travelling distance to clinic**	5–10 km	48	21.9
	Less than 5 km	164	74.8
	More than 10 km	7	3.2
**Duration to reach clinic**	30 min to 1 h	26	11.8
	Less than 30 min	193	88.1
**Type of transport**	Own transport	10	4.57
	Taxi	47	21.4
	Walk	161	73.5
**Education**	Completed grade 12	167	76.2
	Secondary school	52	23.7

**Table 2:** Sexual behavior (n = 219) of participants.

Variable	Response	Frequency (N)	Percentage (%)
**Ever had sex**	No	18	8.22
	Yes	201	91.7
**Age at first sex**	13–17 years	105	47.9
	18–24 years	96	43.8
	Missing values	18	8.2
**Median age at first sex**	17 (IQR: 16–18)		
**First sex planned**	Planned	152	76.0
	Unplanned	47	23.5
	Unplanned/raped	1	0.50
	Missing value	19	0.9
**Age of partner at first sex**	My age	62	28.3
	Older than me	125	57.1
	Younger than me	11	5.0
	Missing value	21	9.5
**Partner employment status at first sex**	Do not know	1	0.50
	Partner was not attending school	15	6.8
	Partner was not working	2	0.9
	Partner was working	51	23.3
	We were both at school	132	60.3
	Missing values	18	8.2

**Table 3:** Sexual relationship (n = 219) of participants.

Variable	Response	Frequency (N)	Percentage (%)
**Current sexual relationship**	No	60	27.4
	Yes	159	72.6
**Present relationship status**	Casual partner	17	10.6
	Steady partner	143	89.9
**Current sexual partner age**	My age	14	8.81
	Older than me	131	82.3
	Younger than me	14	8.81
**Partner employment status**	Attending school	28	17.8
	Employed	104	66.2
	Unemployed	25	15.9
**Period of relationship**	Less than 12 months	3	2.29
	1–2 years	33	25.1
	More than 2 years	95	72.5

**Table 4:** Sexual partners (n = 201) of participants.

Variable	Response	Frequency (N)	Percentage (%)
**Number of partners in the past 12 months**	Had 1 partner	139	88.5
	Had 2 partners	11	7.01
	Had more than 2 partners	7	4.46
**Ever had more than one partner at a time**	No	175	81.7
	Yes	39	18.2
**Ever had more than one partner at a time in the past 12 months**	No	196	92.4
	Yes	16	7.55
**Ever have sex in exchange for money**	No	208	98.5
	Yes	3	1.42
**Ever have sex in exchange for money In the past 6 months**	No	210	99.5
	Yes	1	0.47
**Ever have casual sex with someone who is not a regular partner**	No	174	82.4
	Yes	35	16.5
**Ever have casual sex with someone who is not a regular partner in the past six months**	No	201	95.2
	Yes	10	4.74

**Table 5:** Alcohol uptake (n = 201) by participants.

Variable	Response	Frequency (N)	Percentage (%)
**Did you drink alcohol the last time you had sex?**	No	172	86.8
	Yes	25	12.6
	Do not remember	1	0.51
**Planned last sex**	No	43	20.9
	Yes	162	79.0
**Ever been pregnant**	No	62	32.1
	Yes	131	67.8
**Ever made a girl pregnant**	No	15	57.6
	Yes	11	42.3
**Ever discuss contraceptives with partner**	No	16	9.64
	Yes	150	90.3
**Use of contraceptives with partner in the past 6 months**	No	37	22.4
	Yes	128	77.5

**Table 6:** Response of the participants concerning pregnancy (n = 219).

Variable	Response	Frequency (N)	Percentage (%)
**Chances that you can fall pregnant**	High chance	32	16.4
	Low chance	163	83.5
**The likelihood of making a girl fall pregnant**	Likely	7	36.8
	Never	1	5.26
	Unlikely	11	57.8

**Table 7:** Access to condoms (n = 219) by participants.

Variable	Response	Frequency (N)	Percentage (%)
**Easy access and availability of male condoms for youth in the community**	No	2	0.91
	Yes	217	99.0
**Easy access and availability of female condoms for youth in the community**	No	41	18.7
	Yes	178	81.2
**I could purchase condoms without feeling embarrassed**	No	41	18.7
	Yes	178	81.2
**I could go and get condoms from a public place without feeling embarrassed**	No	38	17.3
	Yes	181	82.6
**I always carry condoms with me should I need one**	No	68	31.0
	Yes	151	68.9
**I feel confident suggesting the use of condoms with a new partner**	No	21	9.13
	Yes	198	90.4
**I could refuse sex if my partner does not want to use a condom**	No	38	17.3
	Yes	181	82.6

**Table 8:** HIV testing practices (n = 219) of participants.

Variable	Response	Frequency (N)	Percentage (%)
**Ever tested for HIV**	No	6	2.74
	Yes	213	97.2
**Tested for HIV/AIDS in the past twelve months**	No	16	7.44
	Yes	197	91.6
**Knowledge of current partner’s HIV status**	No	30	17.8
	Yes	138	82.1
**Ever discussed HIV testing with current partner**	No	15	9.3
	Yes	145	90.6
**Chances of asking current partner to go for an HIV test with them**	Likely	136	85.0
	Unlikely	24	15.0

**Table 9:** Perceptions about STIs and condom use (n = 219).

**Variable**	**Response**	**Frequency (N)**	**Percentage (%)**
A person can have an STI without symptoms in the early stage of the STI	Agree	128	59.2
	Disagree	15	6.94
	Not sure	73	33.8
Chances of getting STIs	High chance	34	19.1
	Low chance	142	79.7
	No	2	1.12
**Variable**	**Response**	**Frequency**	**Percentage**
Worried are about getting HIV/AIDS	Worried	46	22.2
	Very worried	64	30.9
	Not worried at all	97	46.8
**Variable**	**Response**	**Frequency**	**Percentage**
Condom use last time they had sex	Yes	107	51.4
	No	101	48.5
Overall condom use in the past 6 months	I never use condoms	45	22.7
	I always use condoms	61	30.8
	I sometimes use condoms	92	46.4
Ever used a female condom	Yes	9	4.84
	No	177	95.1

**Table 10:** STIs, treatment and partner notification (n = 219).

Variable	Response	Frequency (N)	Percentage (%)
**Ever had STIs in the past twelve months**	No	141	64.3
	Yes	74	33.8
	Missing values	4	1.9
**Treatment received**	No	9	12.1
	Yes	65	87.8
**Partner notified about STIs**	No	9	12.1
	Yes	65	87.8
**Completion of received STIs treatment**	No	7	15.2
	Yes	39	84.7
**Reasons for not completing treatment**	I was cured before the treatment was finished	3	33.3
	Never received treatment	6	66.6
**Sexual activity during treatment**	I abstained	38	79.1
	I used condoms all the time	4	8.33
	I continued having sex without a condom	5	10.4
	Never received treatment	1	2.08

**Table 11:** Participants’ reasons for facility visit.

Reasons for Facility Visit (n = 219)		
Response	Frequency (N)	Percentage (%)
**Accompanying friend or relative**	19	8.72
**Collect ARVs**	9	4.13
**Consultation**	22	10.0
**Dental**	3	1.38
**Pregnancy check up**	59	27.0
**HIV/Blood test**	20	9.24
**Pregnancy/pap smear/contraceptives**	85	38.9
**To fetch a treatment for someone**	1	0.46

## Data Availability

Would be available on reasonable request.
